# Age-Stratified Prevalence of Helicobacter pylori Infection in Children With Recurrent Abdominal Pain: A Prospective Observational Study

**DOI:** 10.7759/cureus.76778

**Published:** 2025-01-02

**Authors:** Jishnu KR, Bikash R Sahu, Mirabai Das, Preetam Nath, Seba Ranjan Biswal, Nirmal K Mohakud

**Affiliations:** 1 Pediatrics, Kalinga Institute of Medical Sciences, Bhubaneswar, IND; 2 Pediatric Medicine, Assumption Hospital, Sultan Bathery, IND; 3 Zoology, Centurion University of Technology and Management, Bhubaneswar, IND; 4 Health and Education, Kalinga Institute of Social Sciences (KISS), Bhubaneswar, IND; 5 Gastroenterology and Hepatology, Kalinga Institute of Medical Sciences, Bhubaneswar, IND; 6 Infectious Disease, Kalinga Institute of Biotechnology, Bhubaneswar, IND

**Keywords:** children, gastritis, h. pylori, rap, rapid urease test, upper gi endoscopy

## Abstract

Background

Recurrent abdominal pain (RAP), often considered functional, is a frequent complaint among pediatric patients. However, the increasing availability of advanced diagnostic tools like upper and lower gastrointestinal endoscopy, tests for *Helicobacter pylori*, and abdominal ultrasound have highlighted many organic causes, including *Helicobacter pylori* (*H. pylori*) infection, whose prevalence in children remains unclear.

Objectives

To determine the age-stratified prevalence of *H. pylori* infection and investigate its role along with other etiologies contributing to RAP in pediatric patients.

Methods

This cross-sectional observational study was conducted between October 2019 and April 2021, involving 60 children aged two to 14 years who met the Rome IV criteria for RAP. Comprehensive evaluations included detailed history-taking, physical examination, and upper gastrointestinal endoscopy with biopsy. Mucosal samples from the antrum were subjected to a rapid urease test (RUT) to detect *H. pylori* infection. The presence of endoscopic abnormalities and their association with positive RUT results were analyzed to identify the underlying causes of RAP.

Results

*H. pylori *infection was identified in 11 (18.3%) children, with the highest prevalence among children aged seven to 12 years (n=34; 56.7%). All biopsy-positive cases showed concordant rapid urease test results (p=0.001). Organic etiologies were identified in 34 (56.7%) patients, with gastritis and gastric ulcers being the most common findings. Other causes included mesenteric lymphadenitis, urinary tract infections, duodenal ulcers, and hiatus hernia. Children with* H. pylori* infections predominantly exhibited endoscopic abnormalities, underscoring its role in RAP.

Conclusion

*H. pylori* infection shows a marked increase with age and is a significant organic cause of RAP. Thorough investigations, including endoscopic evaluations, are essential to uncover organic etiologies. Targeted treatment for *H. pylori* should be prioritized in children over seven years presenting with RAP, emphasizing the need for a systematic approach to diagnosing and managing this condition.

## Introduction

Recurrent abdominal pain (RAP) remains a significant challenge in pediatric practice, being defined in the updated Rome IV criteria as abdominal pain persisting for over two months with at least one weekly episode severe enough to impact the child’s daily activities [1]. RAP is often regarded as functional (nonorganic), but organic causes are identified in approximately 5-10% of cases [2].

The advent of advanced diagnostic modalities, including gastrointestinal (GI) endoscopy, has substantially enhanced the identification of organic etiologies in children with RAP. For example, abdominal ultrasonography can identify abnormalities in up to 10% of children meeting criteria for further investigation [3]. Similarly, innovations in pediatric endoscopy have demonstrated increased diagnostic yields for organic causes of RAP, highlighting the importance of early and precise diagnosis [4].

Epidemiological studies estimate that RAP affects 10-20% of school-aged children globally [4,5]. Despite this prevalence, the diagnostic challenge persists due to RAP's multifactorial etiopathogenesis, encompassing both organic and functional disorders. Organic causes of RAP include *Helicobacter pylori *(*H. pylori*) infection, parasitic infestations, eosinophilic esophagitis, carbohydrate intolerance, irritable bowel syndrome, and urinary tract infections (UTIs) [6,7]. Among these, *H. pylori* infection has emerged as an increasingly recognized contributor, with prevalence rates varying by geography, age, and socioeconomic status.

Clinically, RAP presents as intermittent or continuous abdominal pain, often accompanied by red-flag signs such as weight loss, poor growth, gastrointestinal bleeding, and abdominal tenderness. The presence of these red flags necessitates prompt evaluation using a combination of hematology, biochemistry, imaging studies, and endoscopy [2,5]. Endoscopic findings frequently reveal gastritis, gastric ulcers, or other abnormalities, particularly in cases involving *H. pylori* [3,6].

A comprehensive diagnostic approach should also consider differential diagnoses such as psychogenic factors, carbohydrate malabsorption syndromes, and functional disorders like irritable bowel syndrome (IBS). The incorporation of structured frameworks like the Rome IV criteria aids in distinguishing functional disorders from organic causes [1,7].

Management of RAP is tailored to the underlying etiology. Organic causes like *H. pylori* infection warrant antibiotic eradication therapy, whereas functional causes often benefit from dietary modifications, psychological interventions, and reassurance. Complications of untreated RAP include persistent pain, school absenteeism, and impaired quality of life, underscoring the need for timely and accurate diagnosis [4,6].

In this study, we aimed to evaluate the clinical characteristics and endoscopic findings in children with RAP to assess the prevalence of *H. pylori* infection and identify other organic causes. By adopting a systematic and comprehensive diagnostic approach, we aim to contribute to the improved management of RAP in pediatric populations.

## Materials and methods

The study was conducted as a cross-sectional observational investigation at the Kalinga Institute of Medical Sciences (KIMS), Bhubaneswar, Odisha, over 18 months, from October 2019 to April 2021. Data were collected at a single point in time for each participant during their clinical evaluation to identify the prevalence of *Helicobacter pylori* infection and other etiologies of recurrent abdominal pain (RAP) in the pediatric population. The design ensured real-time clinical, endoscopic, and histopathological data collection, minimizing recall bias. Ethical approval was obtained from the institutional ethics committee (KIIT/KIMS/IEC/118/2019), in adherence to the principles of the Helsinki Declaration.

Data collection

A total of 60 children of either sex, between two and 14 years of age, who fulfilled ROME IV criteria for RAP, both from the outpatient clinic and inpatient wards, were included. The Rome IV criteria for recurrent abdominal pain (RAP) in children require the presence of abdominal pain occurring at least four times per month over at least two months, significantly affecting daily activities, with no evidence of an organic disease after appropriate evaluation [8]. Children with a previous endoscopic diagnosis of a gastrointestinal disorder or critically ill children were excluded from the study. A detailed clinical evaluation was done as per the proforma. Hematological and biochemical tests (complete blood count, C-reactive protein, liver function test, renal function test), urine routine, microscopy, culture, and sensitivity, and stool routine, microscopy, culture, and sensitivity, along with ultrasound sonography (USG) abdomen, were done.

Endoscopic procedure and *H. pylori* test

Upper gastrointestinal endoscopy was performed at the Department of Gastroenterology and Hepatology using an Olympus CLV 190 gastroscope under anesthetic support. For children aged >5 years, a combination of local lignocaine spray, midazolam, and pentazocine was utilized, while for those <5 years, propofol and ketamine (1 mg/kg) were administered under multipara monitoring for vital signs. A rapid urease test (RUT) was performed using a mucosal sample from the antrum.

To further investigate pathological changes, 4-6 gastric antral mucosal samples were taken using biopsy forceps. The specimens were immediately fixed in 10% buffered formalin to ensure the preservation of tissue integrity. Subsequently, the samples were processed routinely, and slides were stained using hematoxylin and eosin (H&E) to evaluate the pathological response to *H. pylori*. This standardized approach to RUT and biopsy handling ensures accuracy and reproducibility.

Statistical analysis

Data were entered and analyzed using an Excel sheet and IBM SPSS Statistics for Windows, Version 21 (Released 2012; IBM Corp., Armonk, New York, United States) [9]. Basic descriptive statistics were performed and displayed as frequency tables.

## Results

The mean age of the children was 10.27 ± 3.07 years. It was observed that five, 21, and 34 (56.7%) of the patients were in the age groups of two to five years, six to 10 years, and 11-15 years, respectively, and had RAP. The majority of children were males (M:F; 1.85:1). In the study, *H. pylori* positivity showed an increasing trend with age, being highest (72.7%) in the 11-15 years age group, followed by 27.3% in the six to 10 years group and 10.9% in the two to five years group. However, the association between age groups and biopsy results was insignificant (chi-square=3.40, p=0.49) (Table 1).

**Table 1:** Age group-wise stratification of H. pylori cases in children aged two to 14 years

Age groups (count % within age group)	Biopsy			
	Positive	Negative	Not done	Total
2- 5 years	0	5	0	5
		10.9%		8.3%
6 to 10 years	3	16	2	21
	27.3%	34.8%	66.7%	35%
11 to 15 years	8	25	1	34
	72.7%	54.3%	33.3%	56.7%
Total	11	46	3	60
Chi-square-3.40; p-value-0.49; not significant				

**Figure 1:**
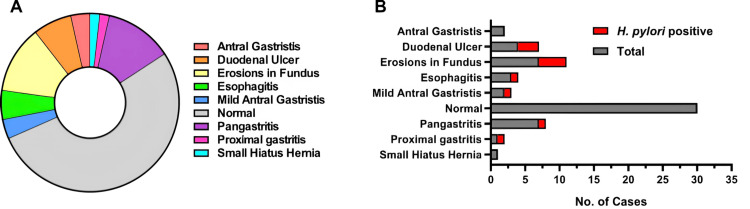
Breakdown of etiological organic causes identified in the biopsy test in children with recurrent abdominal pain (RAP) Panel A: duodenal ulcer, n=4 (6.7%); erosions in fundus, n=7 (11.7%); esophagitis, n=3 (5%); mild antral gastritis, n=2 (3.3%); proximal gastritis, n=1 (1.7%) Panel B: corresponding positive for *H. pylori *cases as per etiology. Duodenal ulcer, n=3 (27.3%); erosions in fundus, n=4 (36.4%)

Figure 1 provides a breakdown of etiological organic causes identified in the biopsy test of children undergoing investigation for recurrent abdominal pain as part of the study on the prevalence of *Helicobacter pylori (H. pylori)*. Panel A shows the distribution of organic causes as a donut chart, highlighting conditions such as antral gastritis, duodenal ulcers, fundal erosions, esophagitis, and other gastric abnormalities. Most cases were classified as normal (n=30, 65.2%), while smaller proportions had diagnoses such as pangastritis (n=6, 13%) or antral gastritis (n=2, 4.3%). Panel B illustrates the corresponding number of cases for each condition, with the red bars representing the subset positive for *H. pylori*. Significant findings include a notable proportion of *H. pylori*-positive cases in antral gastritis and duodenal ulcer groups, demonstrating a possible association between *H. pylori* infection and these conditions.

The majority of patients had symptoms for three to four months, which varies from six months to two years. *H. pylori *was detected in 11 out of 51 normal USG abdomens (p=0.001) (Table 2). It highlights the comparison between USG abdomen findings and biopsy test results in 60 study subjects. Out of 51 patients with normal USG findings, 11 (100%) were biopsy-positive (p=0.001), while 87% were biopsy-negative. None of the patients with acute appendicitis, colitis, mesenteric adenitis, or mild hepatomegaly were biopsy-positive. Similarly, no biopsy positivity was noted in UTI cases. This data underscores the importance of biopsy in diagnosing significant underlying conditions, particularly in cases with normal USG findings, highlighting its role in evaluating pediatric recurrent abdominal pain.

**Table 2:** Comparison of biopsy test according to USG abdomen USG: ultrasound sonography; UTI: urinary tract infection chi-square value-61.6; *p=0.001

	Biopsy			
USG abdomen	Negative	Positive	Not done	Total
Acute appendicitis	0	0	1 (33.3%)	1
Colitis	1 (2.2%)	0	0	1
Mesenteric adenitis	4 (8.7%)	0	0	4
Mild hepatomegaly	1 (2.2%)	0	0	1
Normal	40 (87%)	11^*^ (100%)	0	51
UTI	0	0	2 (66.7%)	2
Total	46	11	3	60

As shown in Table 3, an increased percentage of patients showing positive biopsy results was observed for antral gastritis, duodenal ulcer, erosions in the fundus, and esophagitis diagnosed by gastrointestinal (GI) endoscopy. However, the majority (30, or 65.2%) of negative patients had normal endoscopic findings. All *H. pylori*-positive cases had erosions or gastritis (p=0.001). The rapid urease test showed a positive result in all biopsy-positive cases (p=0.001). Table 3 reveals that the most common diagnosis among patients was idiopathic recurrent abdominal pain, accounting for 26 cases (43.3%). This was followed by pangastritis in six cases (10%), mesenteric adenitis in three cases (5%), and urinary tract infections in two cases (3.3%).

**Table 3:** Comparison of biopsy test according to upper gastrointestinaI endoscopy chi-square value-89.66; *p-value=0.001, biopsy not done in three cases

Upper gastrointestinal endoscopy	Biopsy test		
	Negative	Positive	Total
Antral gastritis	2 (4.3%)	0	2 (3.3%)
Duodenal ulcer	1 (2.2%)	3 (27.3%)	4 (6.7%)
Erosions in fundus	3 (6.5%)	4 (36.4%)	7 (11.7%)
Esophagitis	2 (4.3%)	1 (9.1%)	3 (5%)
Mild antral gastritis	1 (2.2%)	1 (9.1%)	2 (3.3%)
Normal	30 (65.2%)	0	30 (50%)
Pangastritis	6 (13%)	1 (9.1%)	7 (11.7%)
Proximal gastritis	0	1 (9.1%)	1 (1.7%)
Small hiatus hernia	1 (2.2%)	0	1 (1.7%)
Total	46	11^*^	57

## Discussion

The study highlights significant findings regarding recurrent abdominal pain (RAP) in pediatric patients, emphasizing the association between *Helicobacter pylori (H. pylori) *infection and gastrointestinal pathology. Among patients with normal abdominal ultrasonography (USG), 21.6% tested positive for *H. pylori* on biopsy (p=0.001), suggesting its potential role in RAP. Endoscopic findings demonstrated erosive and inflammatory conditions like duodenal ulcers and fundal erosions strongly associated with biopsy positivity (p=0.001), while biopsy-negative cases showed normal endoscopic findings. These findings underscore the importance of targeted diagnostic evaluations in children with RAP.

Pain in the abdomen in children can have multiple etiologies. A significant symptom of *H. pylori* infection includes abdominal pain, accompanied by nausea, loss of appetite, burping, and bloating [10]. The prevalence of *H. pylori *infection has increased, especially in children, possibly due to underdeveloped immunity, improved diagnostics, and lifestyle changes [11-13]. Factors such as contaminated restaurant food and a busy lifestyle contribute to its rising prevalence.

In this study, *H. pylori* infection was most common in children aged seven to 12 years, with prevalence increasing with age from 8.3% in children <5 years to 56.7% in those >11 years. This trend aligns with findings from both developing countries like Saudi Arabia and developed nations like Korea [14-17]. Immune responses, including heightened regulatory T cell activity and less Th17 activity in children compared to adults, may explain age-related differences in infection and inflammation [18].

Of the 60 children studied, 34 (56.7%) had organic RAP, with *H. pylori* being a major contributor. Contrary to earlier findings from India that dismissed a link between *H. pylori* and RAP, this study found a significant association (p=0.001). Children with *H. pylori* infection benefit from a triple-drug regimen (PPI and two antibiotics) for two weeks, alleviating symptoms in 82% of cases [19,20].

The frequency of abdominal pain varied between positive and negative cases. Among *H. pylori*-positive cases, 45.5% experienced pain several times per week, 36.4% once per week, and 18.2% daily, consistent with other studies [21,22]. Upper abdominal pain was most common in positive cases (54.5%) and aligns with earlier findings linking *H. pylori *to epigastric pain [23,24].

Pathological manifestations of *H. pylori*, including gastritis and duodenal ulcers, were evident in endoscopy findings (Figure 1). These results reaffirm the role of *H. pylori* in RAP and highlight the need for diagnostic confirmation through endoscopy and biopsy, particularly in high-prevalence regions [25,26].

Many factors, like dietary habits, socioeconomic status, or environmental conditions, can influence *H. pylori* infection rates. Previous studies have highlighted the significant impact of such variables on *H. pylori* prevalence and associated gastrointestinal disorders, emphasizing the need for their inclusion in future research [27,28]. Addressing these factors will provide a more comprehensive understanding of disease dynamics and strengthen the evidence base for tailored interventions.

Limitations

The study's small sample size (n=60) and cross-sectional design limit its ability to assess causal relationships and long-term outcomes. Factors such as dietary habits, socio-economic status, and environmental exposures were not explored, and the absence of a control group limits generalizability. Future studies with larger samples and longitudinal designs are warranted to validate these findings.

Implications and suggestions

These findings emphasize the necessity of adopting a systematic and evidence-based approach to diagnosing RAP, particularly in older children, where the likelihood of organic causes such as *H. pylori* is higher. Early identification and targeted management of *H. pylori* infection have the potential to significantly alleviate symptoms, improve quality of life, and prevent complications associated with chronic gastritis or gastric ulcers. This study advocates for pediatricians to routinely consider endoscopic evaluations and microbiological testing in children presenting with RAP, especially those with persistent or severe symptoms.

Furthermore, the implications extend beyond clinical diagnosis to broader public health strategies, including addressing dietary habits, improving hygiene practices, and increasing awareness about the potential for treatable organic causes like *H. pylori *infection. Future studies should explore the long-term benefits of eradicating *H. pylori*, assess the role of environmental and socioeconomic factors in infection rates, and establish guidelines for age-specific diagnostic and treatment protocols. 

## Conclusions

Recurrent abdominal pain (RAP) in children remains a challenging condition to diagnose due to its multifactorial etiology, involving both functional and organic causes. Our study highlights the significant role of *Helicobacter pylori (H. pylori)* infection as an under-recognized contributor to RAP, particularly in children aged seven to 12 years. With a prevalence rate of 18.3% in the studied population, *H. pylori* was strongly associated with endoscopic abnormalities such as gastritis and gastric ulcers. Organic causes, including mesenteric lymphadenitis, urinary tract infections, duodenal ulcers, and hiatus hernia, were identified in over half (56.7%) of the children evaluated. Importantly, the use of upper gastrointestinal endoscopy and rapid urease testing demonstrated high efficacy in detecting these underlying organic conditions, reinforcing their role in the diagnostic algorithm for RAP. By integrating advanced diagnostic tools and targeted treatment strategies, we can ensure a paradigm shift in the management of RAP, moving from a symptom-based to a cause-focused approach that will benefit pediatric patients significantly.
